# Identification of Hub Genes Related to Liver Metastasis of Colorectal Cancer by Integrative Analysis

**DOI:** 10.3389/fonc.2021.714866

**Published:** 2021-08-19

**Authors:** Sicheng Liu, Yaguang Zhang, Su Zhang, Lei Qiu, Bo Zhang, Junhong Han

**Affiliations:** ^1^Research Laboratory of Cancer Epigenetics and Genomics, Department of General Surgery, Frontiers Science Center for Disease-Related Molecular Network, State Key Laboratory of Biotherapy and National Clinical Research Center for Geriatrics, West China Hospital, Sichuan University, Chengdu, China; ^2^Research Laboratory of Cancer Epigenetics and Genomics, Department of General Surgery, Frontiers Science Center for Disease-related Molecular Network, West China Hospital, Sichuan University, Chengdu, China

**Keywords:** functional enrichment analysis, hub genes, transcription factor, drug prediction, liver metastasis, colorectal cancer

## Abstract

Liver metastasis of colorectal cancer (LMCRC) severely damages patient health, causing poor prognosis and tumor relapse. Marker genes associated with LMCRC identified by previous study did not meet therapeutic demand. Therefore, it is necessary to identify new biomarkers regulating the metastasis network and screen potential drugs for future treatment. Here, we identified that cell adhesion molecules and peroxisome proliferator-activated receptor (PPAR) signaling pathway were significantly enriched by analyzing the integrated-multiple expression profiles. Moreover, analysis with robust rank aggregation approach revealed a total of 138 differentially expressed genes (DEGs), including 108 upexpressed and 30 downexpressed genes. With establishing protein–protein interaction network, we also identified the subnetwork significantly enriching the metastasis-associated hub genes including ALB, APOE, CDH2, and ORM1. ESR2, FOXO3, and SRY were determined as key transcription factors regulating hub genes. In addition, ADH-1, epigallocatechin, CHEMBL1945287, and cochinchinenin C were predicted as potential therapeutic drugs. Moreover, the antimigration capacity of ADH-1 and epigallocatechin were confirmed in CRC cell lines. In conclusion, our findings not only offer opportunities to understand metastasis mechanism but also identify potential therapeutic targets for CRC.

## Introduction

Colorectal cancer (CRC) is a notorious malignant tumor with high incidence and mortality rate around the world, causing more than 1.9 million new cases and 935,000 deaths in 2020 (1). It was estimated by the World Health Organization that in 2040, CRC would be a dominant public health problem influencing more than 30 million people (2). Liver is the major target organ for metastasis of patients with CRC, leaving patients few treatment alternatives, thus responsible for the majority of cancer-related deaths. If left untreated, the median survival period of patients with liver metastasis is reported to be only 6.9 months, and the 5-year survival rate of unresectable patients is near to 0, while that of patients with liver metastasis completely removed can be up to 30%–50% (3, 4). Therefore, it is preferred to study the molecular mechanism regulating liver metastasis of colorectal cancer (LMCRC), providing evidence for the prevention and new drug screening to improve prognosis and life quality of patients.

Peripheral lymph nodes are considered as the first station of pan-cancer metastasis, followed by distal organ. Widely accepted programs regulating cancer metastasis involve transforming growth factor beta (TGF-beta) and Wnt signaling pathway, and cell adhesion molecules (5–7). Cytokines and chemokines are believed to be secreted by the primary tumor and fertilize distal origin through blood circulation, facilitating metastasis (8, 9). David et al. found progastrin as a potential predictive marker of liver metastasis in CRC by immunohistochemistry (10); other researchers identified HOXD10, SLC13A2, OSM, MMP3, CXCL6, and CXCL8 as liver metastasis-associated hub genes of CRC through bioinformatics (11). Zhang et al. suggested that AMBP, F2, APOH, and other seven hub genes might be related to metastasis (12). Recent studies revealed that the distal metastasis of CRC is regulated by a complex system (13–15), leading to adjuvant therapy and multiple drugs combination (16–18). However, the present therapy could not fully satisfy patients’ demand due to poor prognosis and acute side effect, or failed at clinical trial. Hence, expanding sample sizes, applying novel analysis algorithms to explore new biomarkers, and identifying potential hub genes and related regulating network, like transcription factors, are necessary to understand the mechanism of liver metastasis of CRC. As a result, chemotherapy with a combination of current and new drugs to eliminate tumors is an urgent need.

In this study, we comprehensively analyzed whole expression data of four Gene Expression Omnibus (GEO) series (GSE) containing liver metastasis and primary colorectal cancer by integrated methods and found that cell adhesion molecules and peroxisome proliferator-activated receptor (PPAR) signaling pathway were significantly enriched. Moreover, a total of 138 differentially expressed genes (DEGs), including 108 upexpressed and 30 downexpressed genes were identified by robust rank aggregation. By establishing protein–protein interaction network, ALB, APOE, CDH2, and ORM1 were determined as hub genes; ESR2, FOXO3, and SRY were ascertained as transcription factor regulating hub genes. In addition, ADH-1, epigallocatechin, CHEMBL1945287, and cochinchinenin C were predicted to be potential therapeutic drugs. In addition, we also showed that both ADH-1 and epigallocatechin have significant antimigration capacity against CRC cells *in vitro*. Collectively, this work reveals that these hub genes, transcription factors, and the enriched signaling pathways serve as potential biomarkers for LMCRC.

## Materials and Methods

### Data Download and Quality Control

Matrix data from GSE100480 (19), GSE49355 (20), GSE81558 (21), and GSE41258 (22) were downloaded from the Gene Expression Omnibus (GEO) database. Expression data of GSE100480 and GSE81558 were normalized using robust multichip average (RMA) method; thus, log_2_ transformation not required. GSE49355 was normalized using MAS5 method and GSE41258 using PLIER method before being uploaded; log_2_ transformation were performed to scale these data. Phenodata were investigated, and expression data of liver metastasis (LM) and primary colorectal cancer (PC) were extracted for later analysis. Quantile normalization were applied using R package preprocessCore to ensure the data have the same distribution (23).

### Identification of Differentially Expressed Genes

Least squares, empirical Bayes, and t-test methods based on R package limma were used to analyze the DEGs between LM and PC in four GSEs separately (24). Probes representing multiple genes and duplicated genes were omitted. A *p* < 0.05 and | log_2_fold change (FC) | >1 were defined as the threshold for DEGs screening. Robust rank aggregation method from R package RobustRankAggreg was executed to integrate DEGs from four GSEs (25). Adjusted *p* < 0.05 and | log_2_FC | > 1 were set as the criteria to filter statistically significant DEGs.

### Functional Enrichment Analysis

Gene set enrichment analysis (GSEA) was performed in R package clusterProfiler (26, 27), with genes metric ranked according to average log_2_FC and Kyoto Encyclopedia of Genes and Genomes (KEGG) gene sets from the Molecular Signatures Database (MSigDB) as input. *p* < 0.05 and adjusted *p* < 0.25 were considered as significant.

KEGG pathway and Gene Ontology (GO) of integrated up- and downregulated genes were enriched and annotated by the Database for Annotation, Visualization and Integrated Discovery (DAVID), respectively, including biological process (BP), cellular component (CC), and molecular function (MF) (28, 29). Top terms with *p* < 0.05 were deemed as significant and visualized with R package ggplot2 (30).

### Construction and Analysis of Protein–Protein Interaction Network

Relationships among integrated DEGs were evaluated by STRING (31); interactions with combined score >0.4 were exported to Cytoscape (version 3.8.2, https://cytoscape.org). Nodes’ scores were calculated by plug-in cytoHubba using 12 methods, and the top 50 genes of each method were kept. Genes existing at least 10 out of 12 methods were selected as candidate hub genes.

### Hub Genes Identification With Survival Analysis

The Cancer Genome Atlas (TCGA) colorectal cancer cohort were divided into two groups according to median gene expression level. Gene Expression Profiling Interactive Analysis 2.0 (GEPIA2) was used to evaluate the difference in disease-free survival (DFS) between the groups (32). Mantel–Cox test value <0.05 was set to determine hub genes associated with poor prognosis of colorectal cancer. Comparisons of hub genes’ expression between LM and PC from GSE41258 were analyzed using Wilcoxon rank sum test with continuity correction; *p* < 0.05 were considered as statistically significant.

### Transcription Factors Prediction

The online website oPOSSUM 3.0 was used to predict the transcription factor (TF) of the hub genes (33). Overrepresented conserved TF binding sites were detected based on the criteria that the conservation cutoff was 0.4. The amount of upstream/downstream sequence was 5,000/5,000; the species was *Homo sapiens*, with Z-score value >10. Pearson’s correlation coefficient were used to the determine the relationship between transcription factors and genes; *p* < 0.05 was considered as statistically significant.

### Gene and Potential Drugs Interaction

Protein expression level in different stages of CRC was curated from the Clinical Proteomic Tumor Analysis Consortium (CPTAC) by UALCAN. The immunohistochemical images of the hub genes in advance and early stages were also identified using Human Protein Atlas (34, 35). The Drug Gene Interaction Database (DGIdb) was used to predict the potential drugs targeting hub genes (36). Drugs with combined value of query score and interaction score >10 were selected for docking. Homologous structures of gene-encoded protein were downloaded from the Protein Data Bank (PDB) and three-dimension structures of drug from PubChem (37, 38). Autodock (version 4.2.6) software was used to preprocess and define the proteins and drugs to receptors and ligands, respectively (39). The docking grid box was set to envelop the whole receptors; docking parameters was set as genetic algorithm with short maximum number of evaluations. The docking results were ranked by energy, and the first model was exported to Pymol for visualization (40).

### Transwell Assay

HCT116 and LoVo cells were harvested and resuspended with Dulbecco’s modified Eagle’s medium (DMEM) at a concentration of 3 × 10^6^ cells/ml. Then, 200 μl cell suspension with 200 μM ADH-1 (Cat. No. HY-13541, MedChemExpress, USA) or epigallocatechin (Cat. No. HY-N0225, MedChemExpress, USA) was added into the top chambers, and the bottom chambers were filled with DMEM with 10% fetal bovine serum (FBS). Cells were allowed to migrate for 24 h. Non-migrated cells were erased with a cotton swab, and migrated cells were fixed with 4% paraformaldehyde for 20 min and stained with crystal violet.

### Colony Formation Assay

LO2, Huh7, and SK-Hep1 cells were digested with trypsin to obtain a single cell suspension. Approximately 500 cells were seeded onto a 48-well plate with different concentrations (0, 50, 100, 200, and 400 μM) of ADH-1 or epigallocatechin and incubated for 10 days. When the colony was visible to the naked eye, the colonies were carefully washed twice with phosphate-buffered saline (PBS). Then, the colonies were fixed with 4% paraformaldehyde for 20 min and stained using crystal violet.

### CCK8 Assay

LO2, Huh7, and SK-Hep1 cells were digested with trypsin to obtain a single cell suspension. Approximately 2,000 cells were seeded onto a 96-well plate with different concentrations (0,12.5, 25, 50, 100, 200, 400, and 800 μM) of ADH-1 or epigallocatechin and incubated for 72 h. Ten microliters of CCK8 reagent was added and treated for 3 h; then, the absorbance was measured at 450 nm.

### Statistical Analysis

R 3.6.3 (https://www.R-project.org/) and GraphPad Prism 5 were used for statistical analysis. *In vitro* data were expressed as mean ± SEM, Students’ t-test was used for calculation of *p* values. *** indicates *p* < 0.0001.

## Results

### Schematic Workflow of This Study

**Figure 1:**
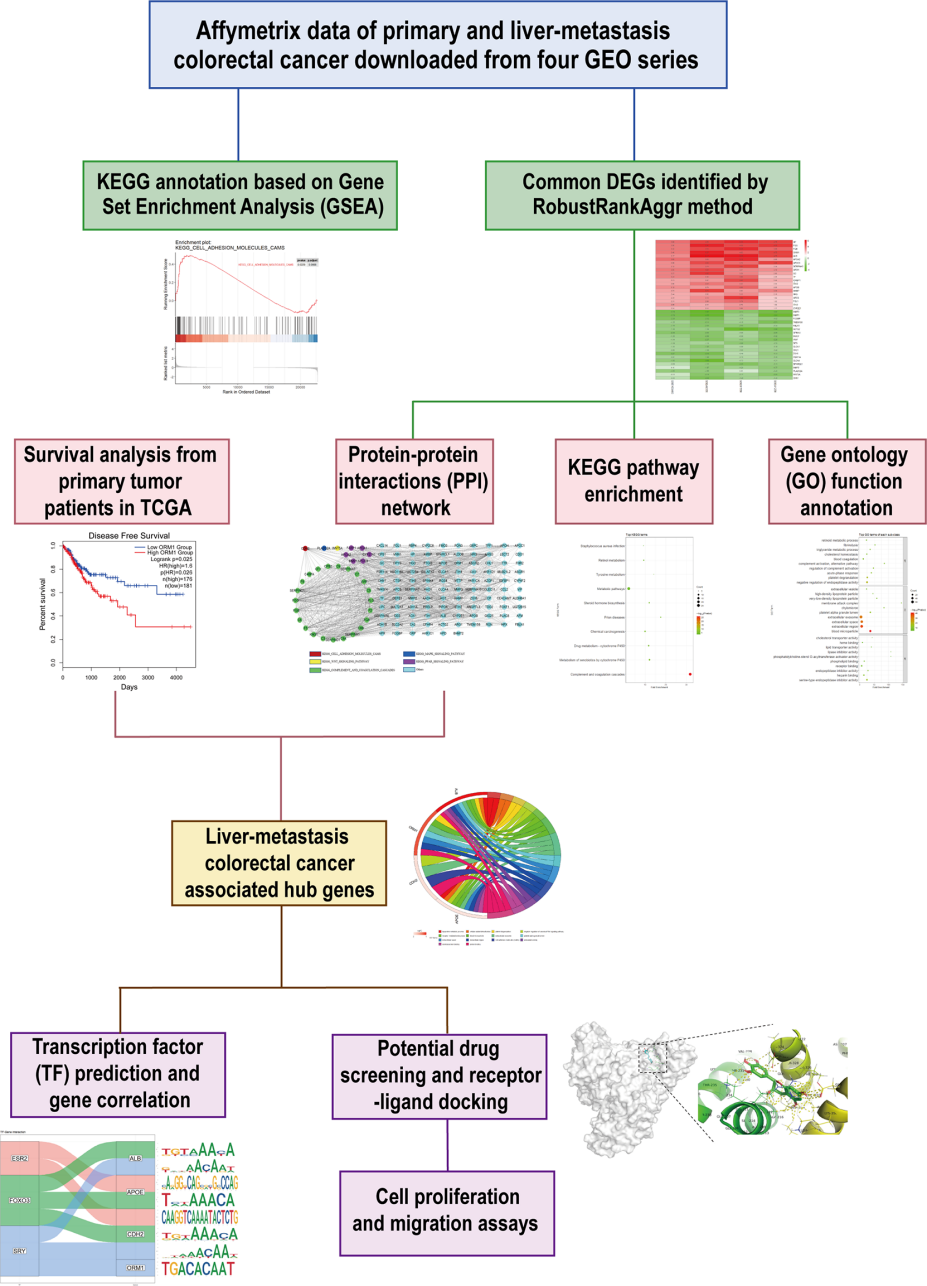
Schematic workflow to present the design of this study.

This study was conducted based on the process described in **Figure 1**. Briefly, we downloaded expression matrix of four GSE series containing liver metastasis (LM) and primary colorectal cancer (PC) from GEO and employed preprocess and quality control to obtain analysis-ready data. GSEA was performed to determine concordant differences between LM and PC. Next, we applied a robust method to integrate and identify common DEGs between LM and PC. To further investigate the characteristic of LM, GO and KEGG annotation were performed. Meanwhile, we constructed a PPI network to analyze the relationships among DEGs. Along with survival analysis from TCGA colorectal patients, we identified four hub genes upexpressed in LM compared with PC, which were associated with poor prognosis of patients. Furthermore, we predicted transcription factor (TF) of hub genes and estimated their correlation. Simultaneously, potential drugs targeting hub genes were predicted, and protein–drug interaction was evaluated. Lastly, *in vitro* experiments were conducted to analyze the antitumor abilities of the two predicted drugs.

### Identification of Differentially Expressed Genes Between LM and PC

**Figure 2:**
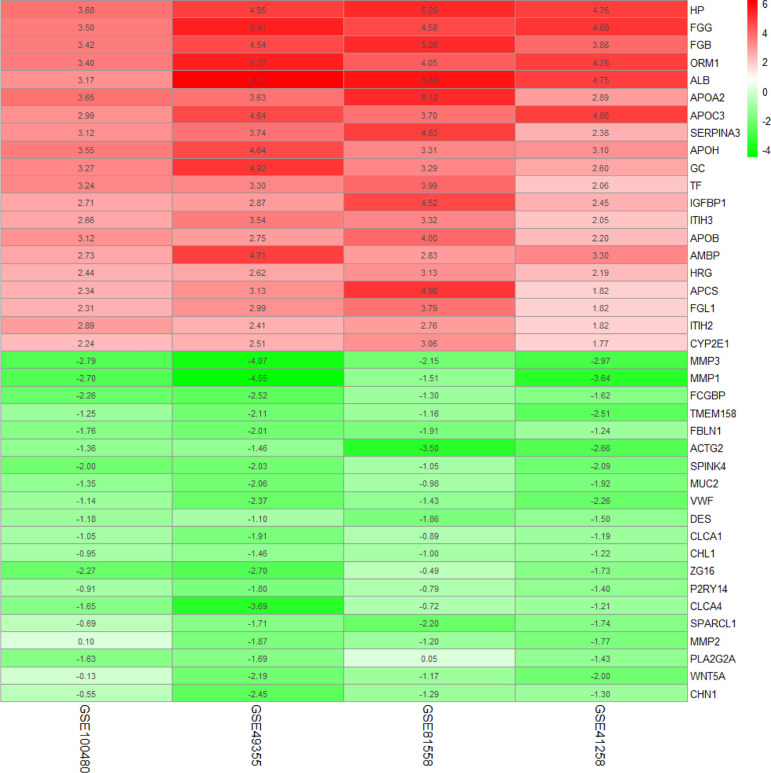
Common DEGs identified by robust rank aggregation algorithm; heatmap shows top 20 of up- and downregulated genes. From green to red, the expression value of the gene in four GEO series gradually increases. The GEO series are presented on the x-axis and gene expression value on the y-axis.

To diminish the system bias in microarray data and make sure that the difference was biologically significant, quantile normalization method was used in the selected sample of four GSEs (**Supplementary Figures S1A–D**). There were 8 LM and 13 PC in GSE100480, 19 LM and 20 PC in GSE49355, 19 LM and 23 PC in GSE81558, and 47 LM and 186 PC in GSE41258; a total of 93 liver metastasis tumor and 242 primary tumor expression data were included in this study. Limma identified 336 significantly upexpressed and 83 downexpressed genes in GSE100480 (**Supplementary Figure S2A**), 171 upexpressed and 127 downexpressed genes in GSE49355 (**Supplementary Figure S2B**), 189 upexpressed and 69 downexpressed genes in GSE81558 (**Supplementary Figure S2C**), and 88 upexpressed and 102 downexpressed genes in GSE41258 (**Supplementary Figure S2D**). Heatmaps demonstrating expression of DEGs were also shown (**Supplementary Figures S3A–D**). Robust rank aggregation method was executed to integrate DEGs from the four GSE series; eventually, 138 overlapping genes including 108 significantly upexpressed and 30 downexpressed genes were obtained (**Figure 2** and **Supplementary Table 1**).

### GSEA, GO, and KEGG Annotation Revealed Distinct Characteristic of LM

**Figure 3:**
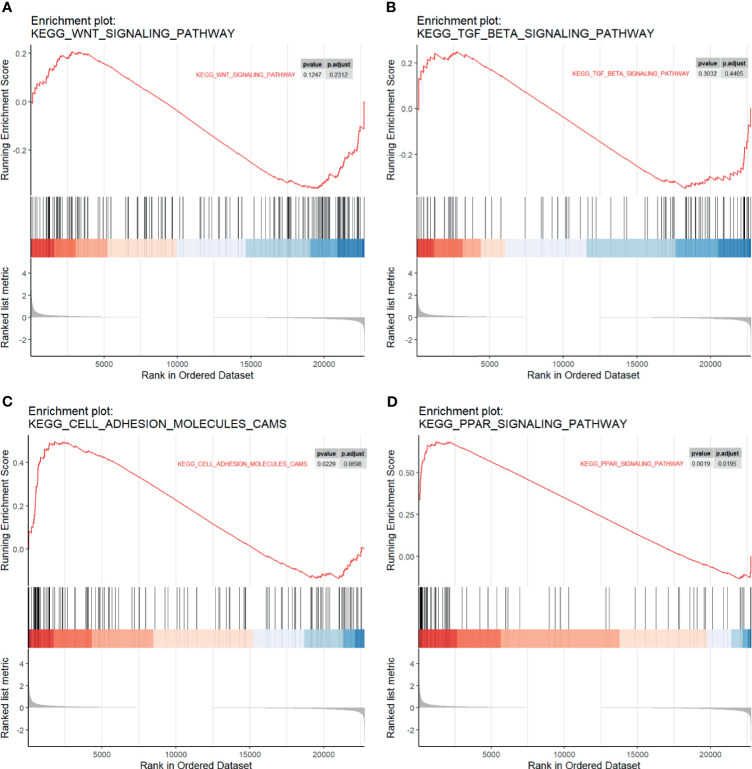
Gene set enrichment analysis (GSEA) plots. The enrichment of the **(A)** Wnt pathway, **(B)** TGF-beta pathway, **(C)** cell adhesion molecules, and **(D)** PPAR pathway in different genes among four GSE series. Ranked list metrics were determined by log2(fold change).

We performed GSEA to investigate whether *a priori* defined gene sets relevant to cancer metastasis were significantly different between LM and PC. A total of 22,750 genes were involved. Results suggested that genes comprising the Wnt signaling pathway (**Figure 3A**) and TGF-β (**Figure 3B**), two canonical pathways widely accepted for regulating metastasis of cancer, were not statistically significant. However, we observed significant enrichment in cell adhesion molecules (**Figure 3C**) and PPAR signaling pathway (**Figure 3D**), which may participate in the regulatory role of liver metastasis.

To explain the biological difference between LM and PC groups, functional enrichment of integrated DEGs was also performed. According to the results, upexpressed genes were significantly enriched in the negative regulation of endopeptidase activity of BP, blood microparticle of CC, and serine-type endopeptidase inhibitor activity of MF (**Supplementary Figure S4A**). Besides, enriched KEGG pathway of upexpressed genes were significant in complement and coagulation cascades (**Supplementary Figure S4B**). In addition, the downexpressed genes were mainly associated with the collagen catabolic process of BP, proteinaceous extracellular matrix of CC, and metalloendopeptidase activity of MF (**Supplementary Figure S4C**). Meanwhile, KEGG enrichment of downexpressed genes were significantly participated in pancreatic secretion (**Supplementary Figure S4D**).

### Hub Genes Associated With Poor Prognosis of Colorectal Patients

**Figure 4:**
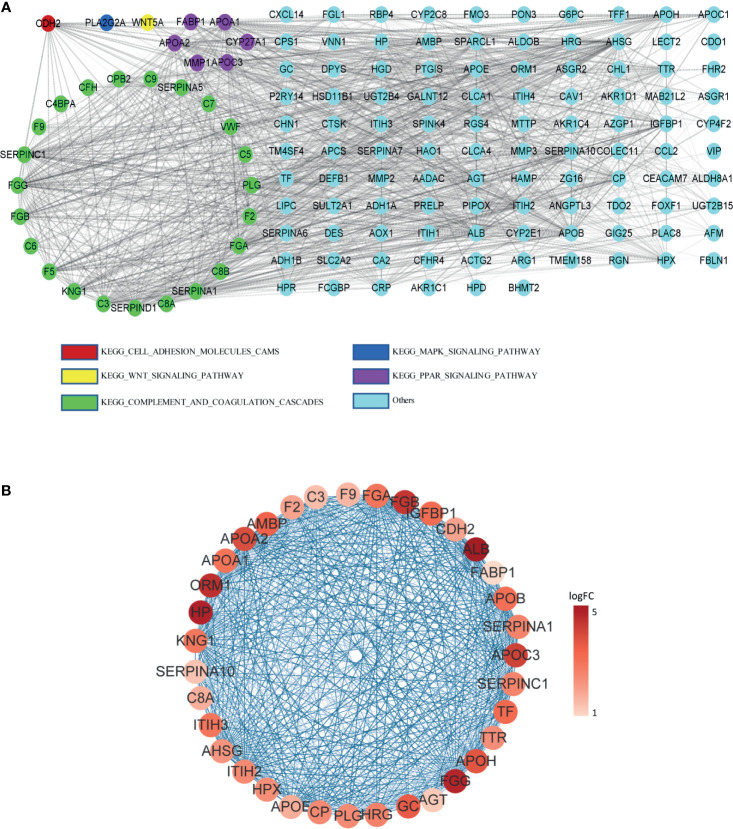
PPI network among DEGs. **(A)** PPI network constructed by STRING; DEGs involving in KEGG pathway were represented by different colors. **(B)** Candidate hub genes with interactions visualized in Cytoscape; from pale red to dark red, the log2FC value of the gene in the sample gradually increases. Edge between two nodes indicates interactions.

**Figure 5:**
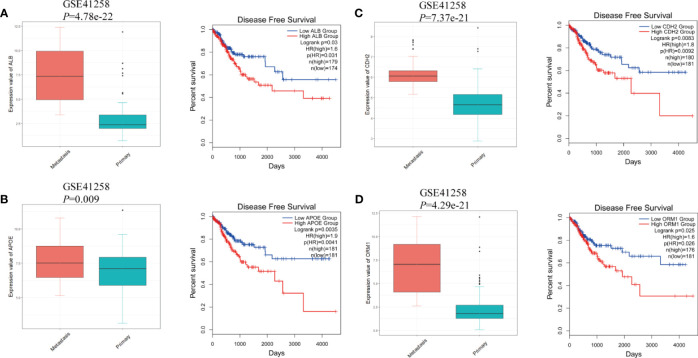
The gene expression at different regions of colorectal cancer in GSE41258 and survival analysis from GEPIA2. **(A)** ALB, **(B)** APOE, **(C)** CDH2, and **(D)** ORM1. The statistical significance of correlations was determined using Wilcoxon rank-sum test with continuity correction and Mantel–Cox test, respectively.

The protein–protein interaction network of DEGs in LM was constructed *via* STRING. Except for combined score <0.4, a total of 138 nodes and 1,388 edges were established in Cytoscape (**Figure 4A**). A node represents a gene encoded protein, and an edge indicates mutual interaction. Plug-in cytoHubba was used to calculate nodes’ scores with 12 methods, and 35 nodes with 506 edges were selected as candidate hub genes (**Figure 4B** and **Supplementary Table S2**), which were all remarkably upexpressed in the LM group (**Figures 5A–D**). We next looked into DFS information of TCGA CRC cohort using GEPIA2. High expression of ALB (**Figure 5A**), APOE (**Figure 5B**), CDH2 (**Figure 5C**), and ORM1 (**Figure 5D**) were identified as biomarkers significantly related to poor prognosis of CRC patients. GO and KEGG analysis of these four genes indicated that they were responsible for extracellular exosome and protein binding (**Supplementary Figure S5**). The above three characteristics illustrated that these four hub genes contribute to LM and severely threat patients’ health.

### Correlation Between Predicted Transcription Factors and Hub Genes

**Figure 6:**
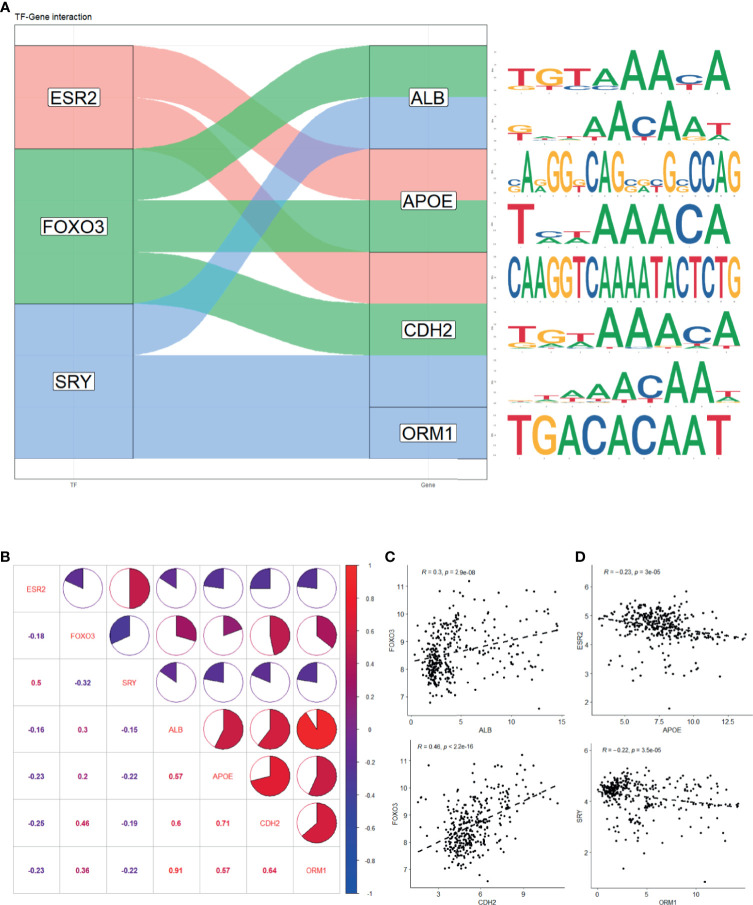
Prediction of hub genes-related transcription factor. **(A)** Potential binding site of TF and genes predicted with oPOSSum 3.0. **(B)** Expression correlation of TF and genes in four GEO series. **(C)** Significant positive correlation between ALB and FOXO3 expression and CDH2 and FOXO3 expression. **(D)** Significant negative correlation between APOE and ESR2 expression and ORM1 and SRY expression. The statistical significance of correlations was determined using Pearson’s correlation coefficient.

To further investigate the underlying mechanism that regulates hub genes, potential transcription factors (TFs) were predicted by oPOSSUM 3.0 (**Supplementary Table S3**). Based on prescribed criteria, we obtained three TFs, namely, ESR2, FOXO3, and SRY. The estimated binding site of TFs and genes was established, respectively (**Figure 6A** and **Supplementary Table S4**). We then examined whether an association existed between TFs and hub genes in samples recruited in this study (**Figure 6B**). There was indeed a positive correlation between ALB and FOXO3 expression and that of CDH2 and FOXO3 expression (**Figure 6C**). We also found a negative correlation between APOE and ESR2 expression and ORM1 and SRY expression (**Figure 6D**). Interestingly, the four hub genes were strongly positively correlated with each other (*R* > 0.5), implying that they might have synergistic effect to promote LMCRC.

### Screening of Potential Drugs for LM Patients

**Figure 7:**
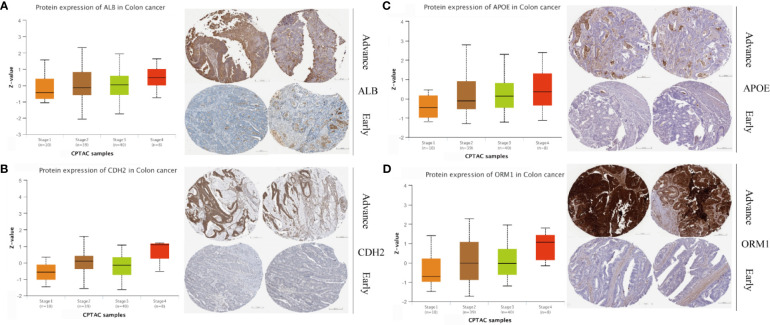
Protein expression level in different stages of colon cancer. Protein expression and immunohistochemical images of **(A)** ALB, **(B)** CDH2, **(C)** APOE, and **(D)** ORM1. Data were extracted from UALCAN and Human Protein Atlas, respectively.

**Figure 8:**
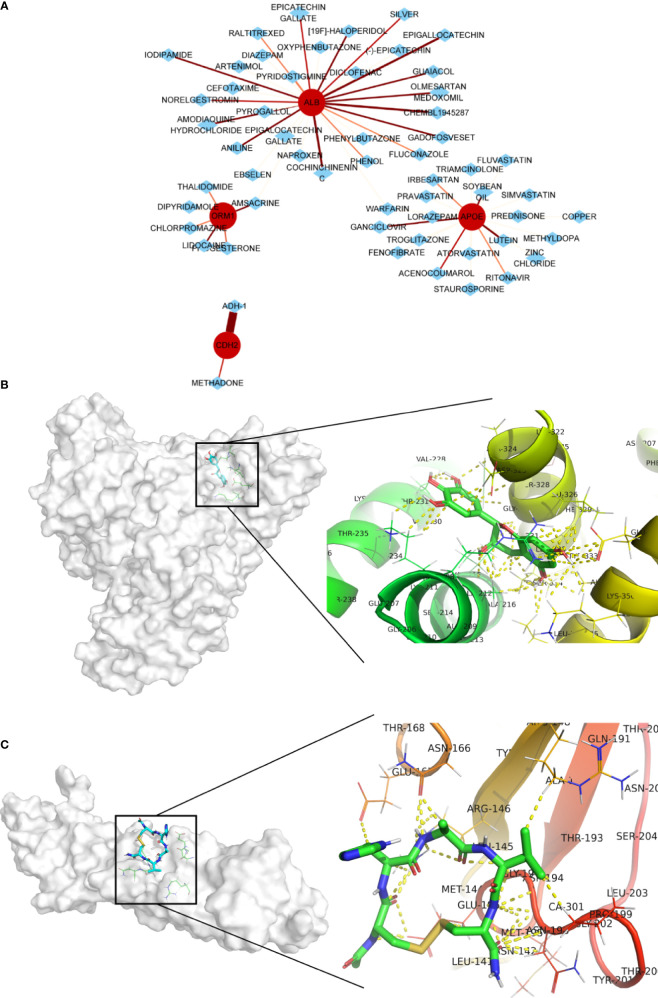
Potential drug screening of hub genes and interactions. **(A)** Drug prediction using DGIdb online database; the size and color of line indicates interaction score. Protein–drug interactions between **(B)** ALB and epigallocatechin and **(C)** CDH2 and ADH-1. Yellow dot indicates any type of interactions.

Four genes, namely, ALB, APOE, CDH2, and ORM1, were considered as hub genes and highly expressed in LM. We examined protein expression of these genes and found that higher expression accompanied with advanced stage (**Figures 7A–D**). It was consistent with our previous findings. Thus, hub genes might serve as potential therapeutic targets. In this context, we used DGIdb online database to search drug–gene interactions. A total of 58 drugs targeting hub genes were obtained (**Figure 8A** and **Supplementary Table S5**). Among them, four drugs, namely, ADH-1, epigallocatechin, CHEMBL1945287, and cochinchinenin C, receiving higher scores than preset criteria were selected. The molecular structures of drugs and proteins downloaded from PubChem (CID: 9916058, 72277, 18877472, and 23634528) and PDB (6RJV and 2QVI) were demonstrated (**Supplementary Figures S6A–F**) separately. Docking results showed that interactions existed between ALB and epigallocatechin (**Figure 8B**) with binding energy of −3.89, CDH2 and ADH-1 (**Figure 8C**) with binding energy of −5.04, ALB and cochinchinenin C (**Supplementary Figure S7A**) with binding energy of −2.32, and ALB and CHEMBL194528 (**Supplementary Figure S7B**) with binding energy of −3.9, respectively. These drugs were potentially prospective agents to prevent and treat LM by interfering with hub genes ALB and CDH2.

**Figure 9:**
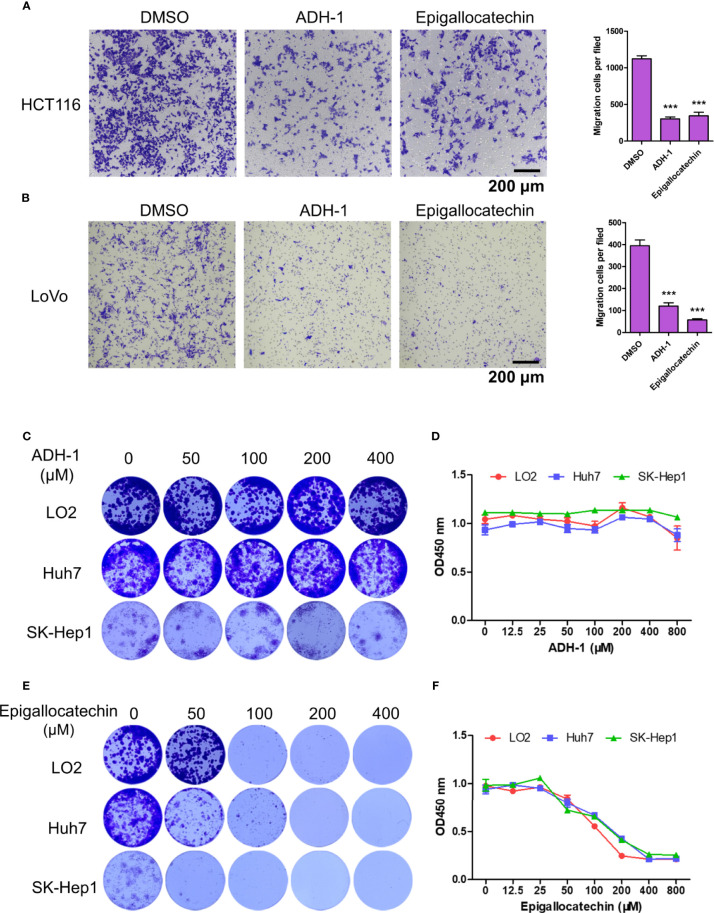
ADH-1 and epigallocatechin suppress colorectal cancer cells metastasis. Transwell assay for **(A)** HCT116 and **(B)** LoVo treated with ADH-1 and epigallocatechin at 200 μM, 24 h. Left panel: crystal violet staining. Scale bar = 200 μm. Right panel: statistic results of migration cells per filed (n = 6). ***p < 0.0001. Colony formation assay for LO2, Huh7, and Sk-Hep1 treated with **(C)** ADH-1 and **(E)** epigallocatechin for 10 days at different concentrations. CCK8 kit measured cell viabilities of LO2, Huh7, and SK-Hep1 treated with **(D)** ADH-1 and **(F)** epigallocatechin for 3 days at different concentrations (n = 6). Statistics shown as mean ± SEM.

To further confirm our findings, *in vitro* experiments were conducted with two available drugs. First, the capacity of ADH-1 and epigallocatechin on colorectal cancer metastasis was examined. The transwell assay results showed that both ADH-1 and epigallocatechin significantly inhibited the migration of HCT116 and LoVo cells (**Figures 9A, B**). Meanwhile, we also evaluated whether ADH-1 and epigallocatechin had an effect on the proliferation of normal liver cell LO2 and tumor cells such as Huh7 and SK-Hep1. As we expected, colony formation and CCK8 indicated that ADH-1 did not affect the proliferation of normal liver cells and tumor cells (**Figures 9C, D**), while epigallocatechin reduced liver cells’ proliferation abilities (**Figures 9E, F**). These results supported that both potential drugs can prevent colorectal cancer cells metastasis.

## Discussion

LMCRC is the major cause for poor prognosis and tumor recurrence. In the present study, gene expression data of four GSE series containing LM and PC were included for comprehensive analysis. A total of 138 liver metastasis-associated DEGs of CRC were identified from four GSE series using robust rank aggregation (RRA) method. Among them, 108 genes were upexpressed and 30 downexpressed. The GO and KEGG enrichment results of DEGs suggested that the metastasis process was a complex system involving a variety of function change. Molecules binding activity, organelle formation, and cell metabolism somehow contributed to cancer metastasis. Ayuko et al. suggested that exosomes may play a critical role in metastasis of cancer cells in the body, which was consistent with our findings (41). By constructing protein–protein interaction network, we observed vast mutual interactions among DEGs, indicating their complementary function. Disease-free survival is defined as the time from randomization to recurrence of tumor or death; therefore, it is a better criterion than overall survival in this study. Taking gene scores and survival analysis into consideration, we then identified four hub genes, namely, ALB, APOE, CDH2, and ORM1, associated with liver metastasis and poor outcome. Albumin (ALB) is a protein-coding gene, and its major function is binding water and irons. Shen et al. suggested that it could be used as an indicator of the metastasis risk of bladder malignant tumors (42). Apolipoprotein E (APOE) is a protein associating with lipid particles. It can bind to a specific liver and peripheral cell receptor. Hyo et al. found that APOE was a useful marker for assessing nonsmall cell lung cancer (NSCLC) patients with lymph node metastasis (43). N-cadherin (CDH2) is a classical cadherin and is related to cell adhesion. The loss of E-cadherin expression and upregulation of N-cadherin, which is called cadherin switch, were well investigated and universally acknowledged as a marker for tumor metastasis progression (44). Orosomucoid 1 (ORM1) encodes a key acute phase plasma protein. However, the specific function of this protein has not yet been determined. We assume that it cooperates in inflammation and promotes cancer metastasis by reprogramming and changing the immune microenvironment.

Based on the average log_2_FC of genes among four GSE series, we performed GSEA to explore the concordant differences between the two stages. The results suggested that PPAR signaling pathway, instead of Wnt or TGF-beta signaling pathway, along with cell adhesion molecules, was significantly enriched in liver metastasis stage. There were five upregulated genes, namely, APOA1, APOA2, APOC3, CYP27A1, and FABP1 in DEGs, and one downregulated gene, MMP1, which belongs to PPAR signaling pathway that contains three ligand-activated transcription factors, PPARA, PPARD, and PPARG. Unsurprisingly, there was a line of evidence to support our findings that activating PPARA could reduce metastatic nonsmall cell lung cancer growth (45) and the protective role of PPARD in melanoma metastasis (46). In addition, a high correlation between PPARD expression and metastasis-free survival is demonstrated experimentally and clinically (47). The previous study has shown that PPARG could promote metastasis in prostate cancer cells (48). In other words, PPAR signaling pathway participated in cancer metastasis through crosstalk with other pathways. Meanwhile, our findings also indicated that further studies on its role in LMCRC are also needed.

To investigate in-depth the underlying mechanism regulating hub genes, we predicted their TFs and theoretical binding sites. The expression results illustrated that ESR2, FOXO3, and SRY jointly regulates hub genes. The correlation between TF and genes showed that FOXO3 positively regulated ALB and CDH2, and ESR2 and SRY negatively regulated APOE and ORM1, respectively. FOXO3 potentially regulated three hub genes and positively correlated with all hub genes, which might serve as a therapeutic target to treat LMCRC. However, current studies did not explain the interactions well; further evidence(s) to uncover the regulating networks is still needed. We also found strong autocorrelations among the four hub genes, suggesting that a synergistic effect contributes to reprogramming of the tumor microenvironment. All of the above serve as evidence to design TFs or hub genes-targeted drugs to prevent and treat LMCRC.

Consequently, we predicted potential drugs and focused on four drugs with higher scores. Adherex’s biotechnology compound (ADH-1) has been used to treat prostate and pancreatic cancer (49, 50). There were several clinical trials about ADH-1 treatment against locally advanced or metastasis pancreatic or biliary tract cancer ((NCT01825603, phase I) and solid tumors (NCT00265057, phase II). ADH-1 can directly target at N-cadherins expressed in cancer cells to disturb cadherin-mediated signaling transduction, eventually leading to apoptosis of cancer cells or causing angiolysis and damage to tumor cells. Thus, it is reasonable that in our study, ADH-1 significantly inhibited colorectal cancer cells migration but had no obvious effect on growth of liver cells possibly due to scarcity of N-cadherins. Epigallocatechin is a flavan-3-ol containing a benzopyran-3,5,7-triol linked to a 3,4,5-hydroxyphenyl moiety and has completed phase II clinical trials with the purpose of treating prostate cancer (NCT00669656). Epigallocatechin showed great effect in inhibiting colorectal and liver tumor cells migration and proliferation. However, its nonspecific cytotoxicity effect on normal liver cells may attenuate the advantages of clinical treatment. We retrieved no existing information about cancer treatment with CHEMBL1945287 and cochinchinenin C. Nevertheless, the potential anticancer properties of these drugs, especially combined with other approved treatment, are promising to relieve the burden of patients with LMCRC.

In this study, we validated existing evidence and proposed new mechanism regulating LMCRC, potential therapeutic targets, and prospective drugs using bioinformatics, providing an avenue for better healthcare. However, limitations still remain to be solved, and the results shown above also need to be validated further by more *in vitro* and *in vivo* approaches in the future.

## Data Availability Statement

Publicly available datasets were analyzed in this study. These data can be found here: GSE100480 (https://www.ncbi.nlm.nih.gov/geo/query/acc.cgi?acc=GSE100480), GSE49355 (https://www.ncbi.nlm.nih.gov/geo/query/acc.cgi?acc=GSE49355), GSE81558 (https://www.ncbi.nlm.nih.gov/geo/query/acc.cgi?acc=GSE81558), GSE41258 (https://www.ncbi.nlm.nih.gov/geo/query/acc.cgi?acc=GSE41258).

## Author Contributions

Conceptualization: SL and JH. Original draft writing and editing: SL, YZ, LQ, BZ, and JH. Figure creation: SL, YZ, and SZ. All authors contributed to the article and approved the submitted version.

## Funding

This work was supported by the National Key Research and Development Program of China (2018YFC1312300), the Foundation for Innovative Research Groups of the National Natural Science Foundation of China (81821002), National Clinical Research Center for Geriatrics (Z20201007), and 1·3·5 Project for Disciplines of Excellence, West China Hospital (ZYGD18003), Sichuan University.

## Conflict of Interest

The authors declare that the research was conducted in the absence of any commercial or financial relationships that could be construed as a potential conflict of interest.

## Publisher’s Note

All claims expressed in this article are solely those of the authors and do not necessarily represent those of their affiliated organizations, or those of the publisher, the editors and the reviewers. Any product that may be evaluated in this article, or claim that may be made by its manufacturer, is not guaranteed or endorsed by the publisher.
