# Supplementary material for: Identification of Hub Genes Related to Liver Metastasis of Colorectal Cancer by Integrative Analysis
Source: Front Oncol. 2021 Aug 19;11:714866. doi: 10.3389/fonc.2021.714866 (PMC8417325; doi:10.3389/fonc.2021.714866)
Supplement: Supplementary file 1 [file DataSheet_1.docx]

**Supplementary Data**


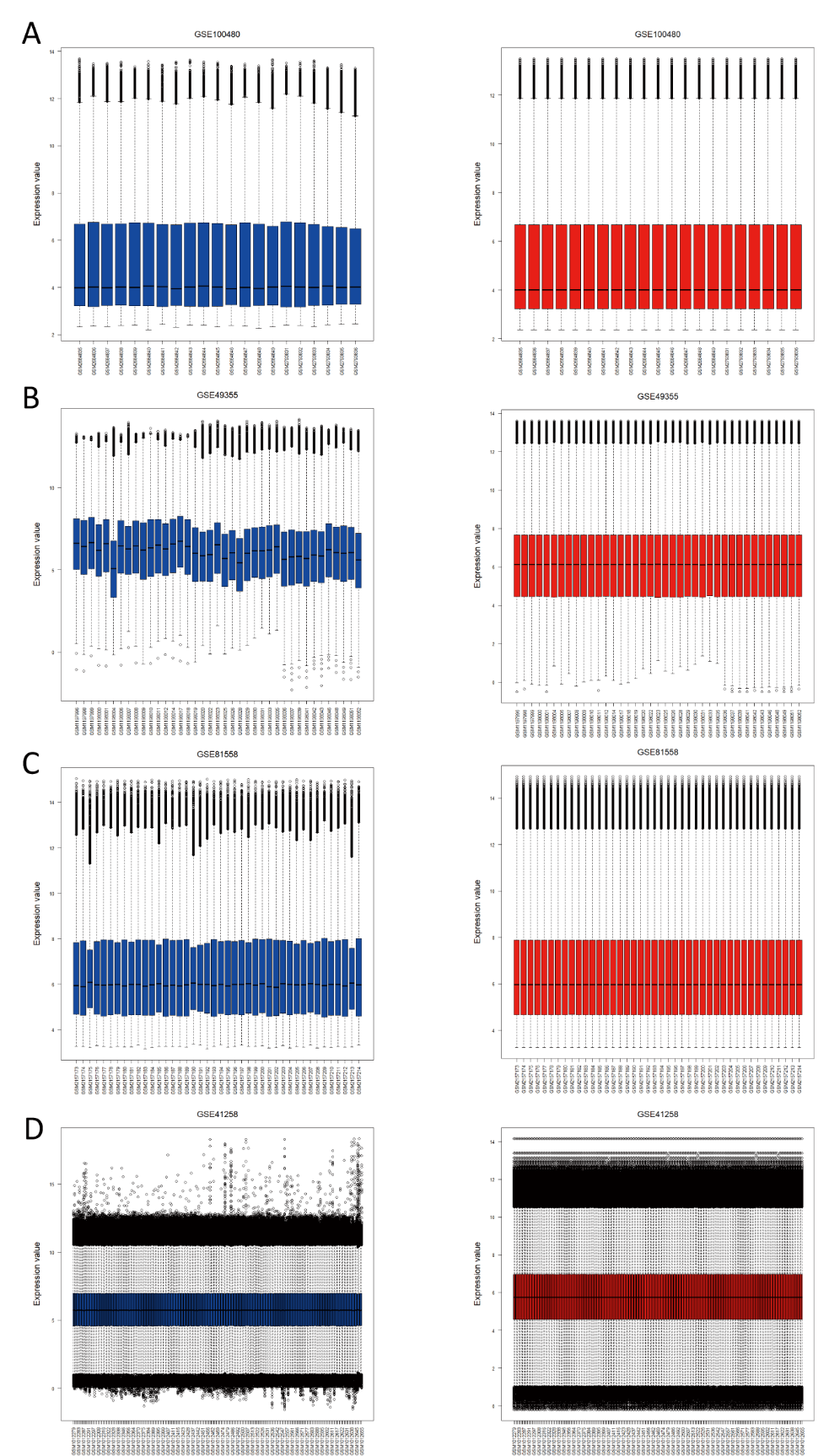


**Figure S1.** Microarray data pre-process using quantile normalization method. Distribution of expression value before normalization of (A) GSE100480, (B) GSE49355, (C) GSE81558 and (D) GSE41258 are shown in blue box, after normalization are shown in red. The sample is presented on the x-axis and gene expression value on the y-axis.


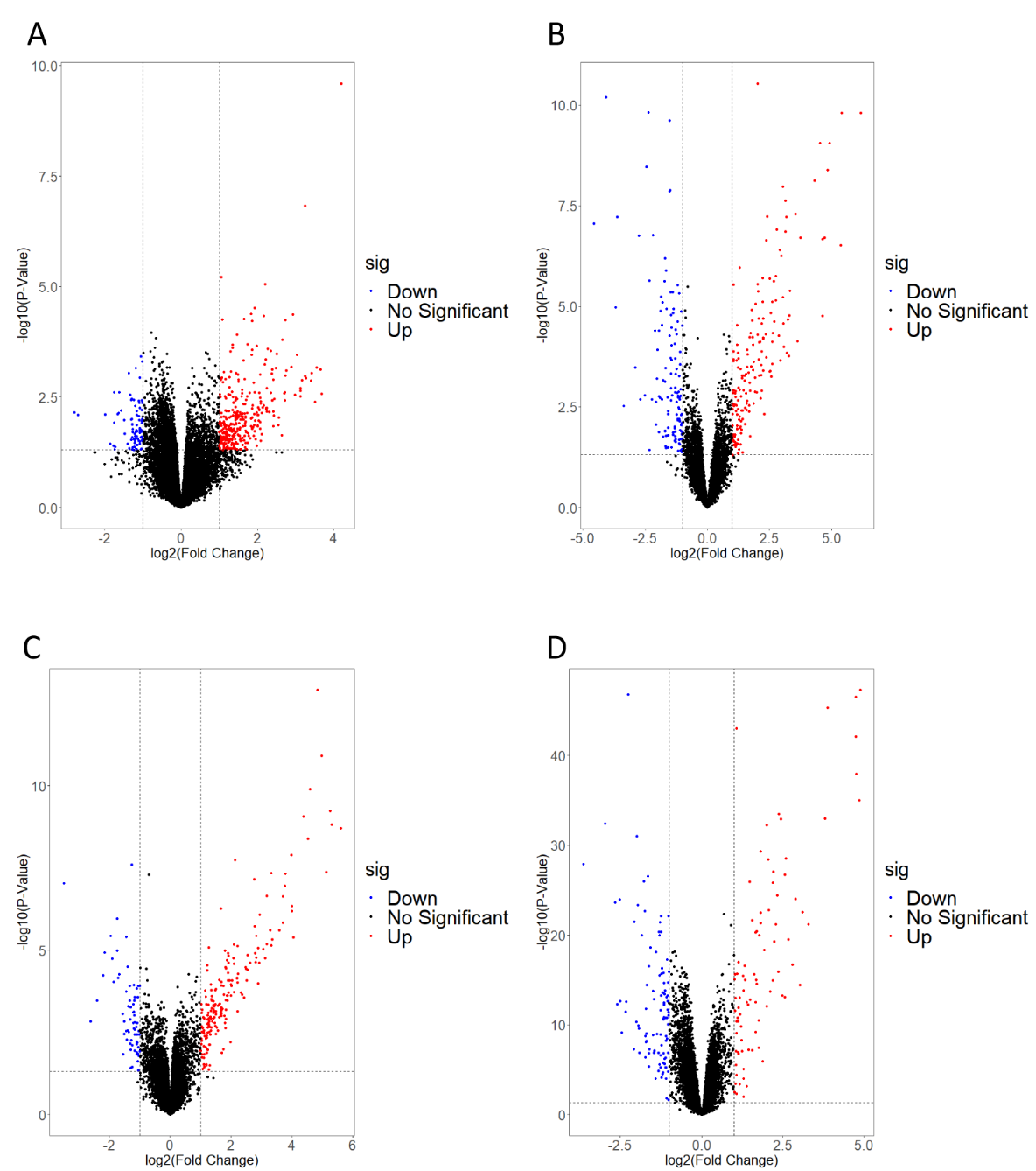


**Figure S2.** Identification of differentially expressed genes. Volcano plot of DEGs in (A) GSE100480, (B) GSE49355, (C) GSE81558 and (D) GSE41258. Red point represents significant up-regulated gene, blue point represents significant down-regulated gene, black point represents gene with no significance. Genes are screened based on *p* value <0.05 and |log2(fold change)|>1.


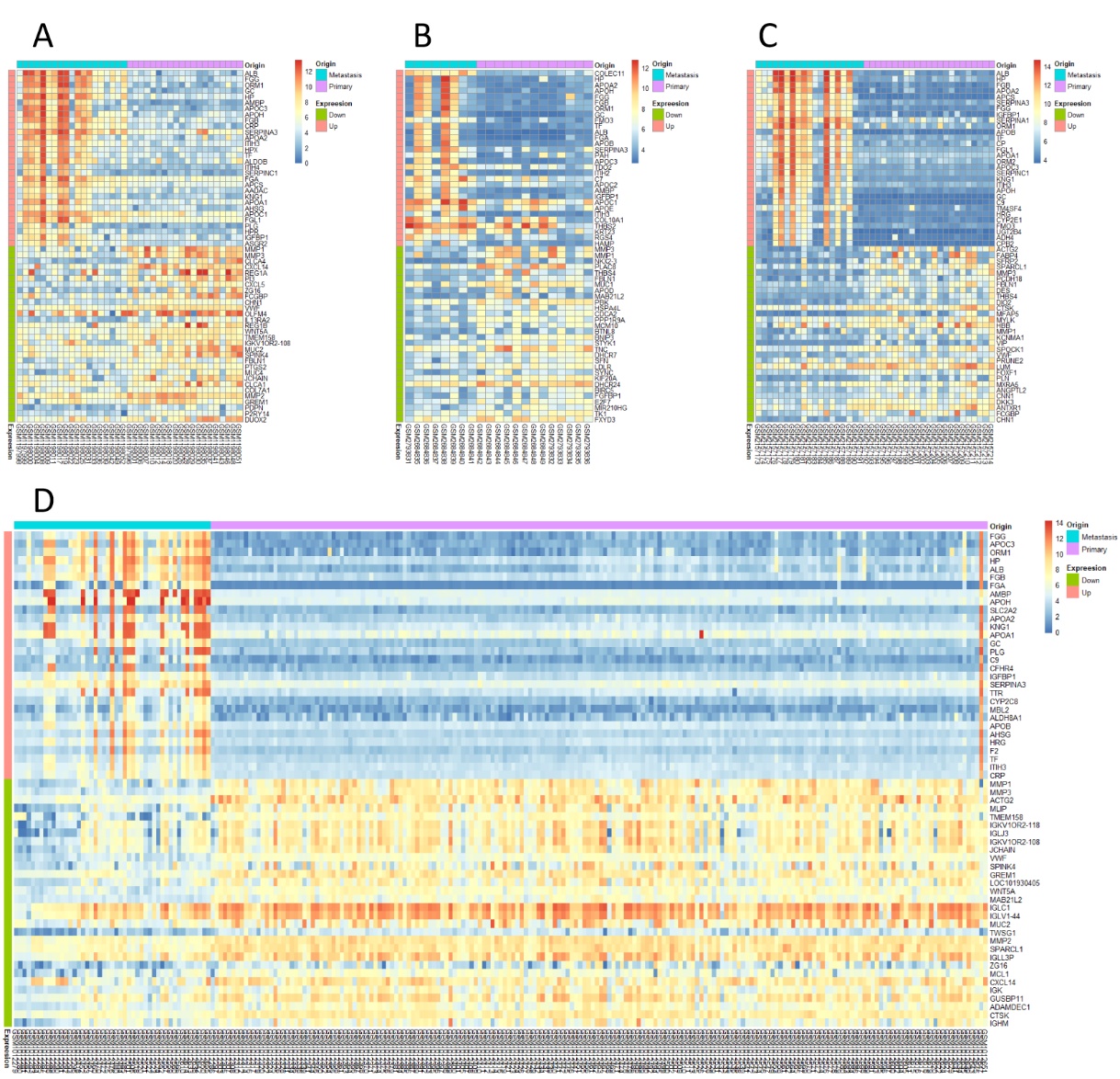


**Figure S3.** Identification of differentially expressed genes. Heatmap of top 30 genes for up and down-regulated genes in (A) GSE100480, (B) GSE49355, (C) GSE81558 and (D) GSE41258. From blue to red, the expression value of the gene in the sample gradually increases. The sample is presented on the x-axis and gene expression value on the y-axis.


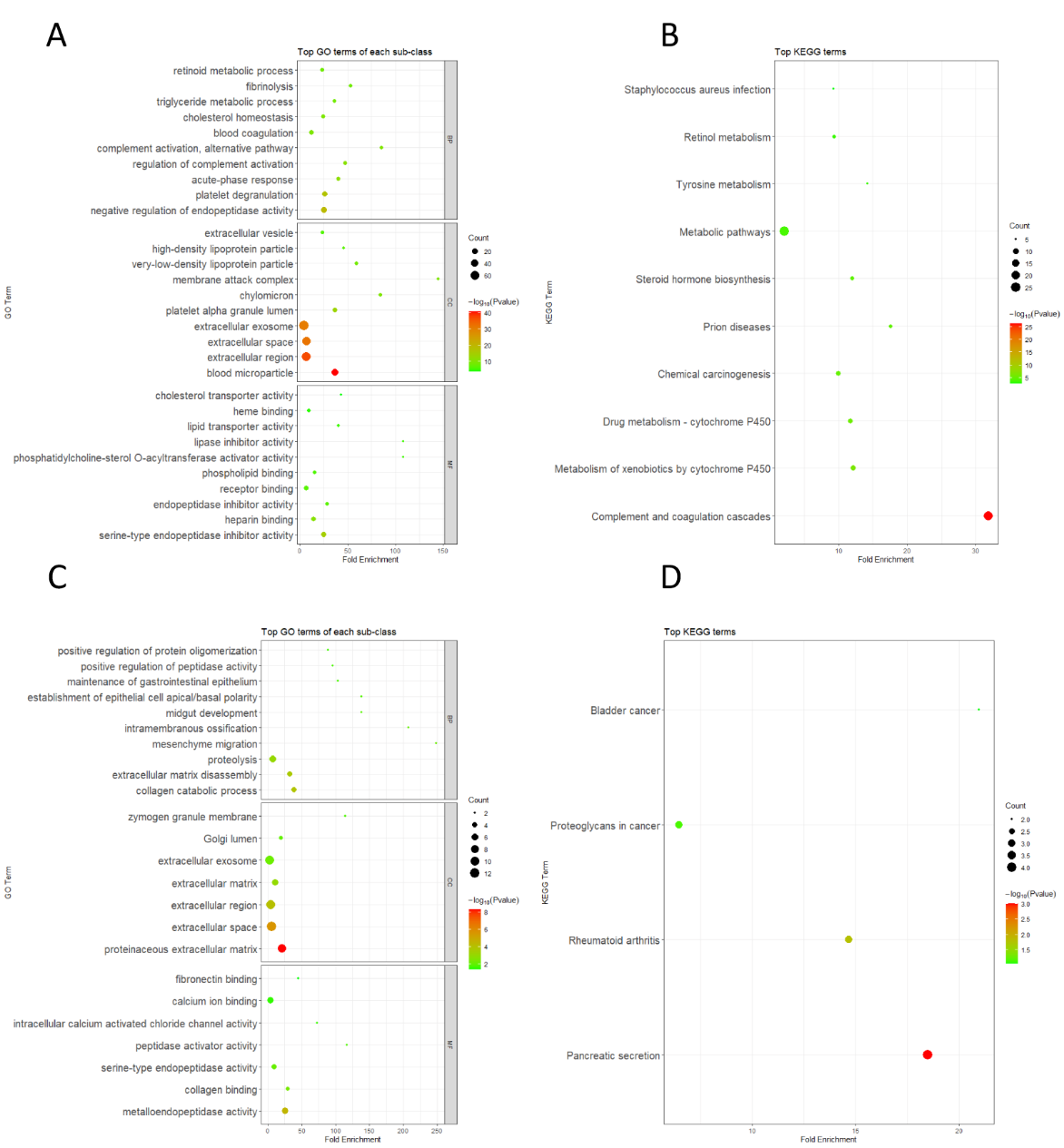


**Figure S4.** Functional enrichment of DEGs. (A, B) The GO and KEGG of up-regulated DEGs respectively. (C, D) The GO and KEGG of down-regulated DEGs respectively. The size of dot represents the number of genes involving in terms, from green to red, the value of –log10(*P*value) gradually increases DEGs, differentially expressed genes; GO, gene ontology; KEGG, Kyoto Encyclopedia of Genes and Genomes.


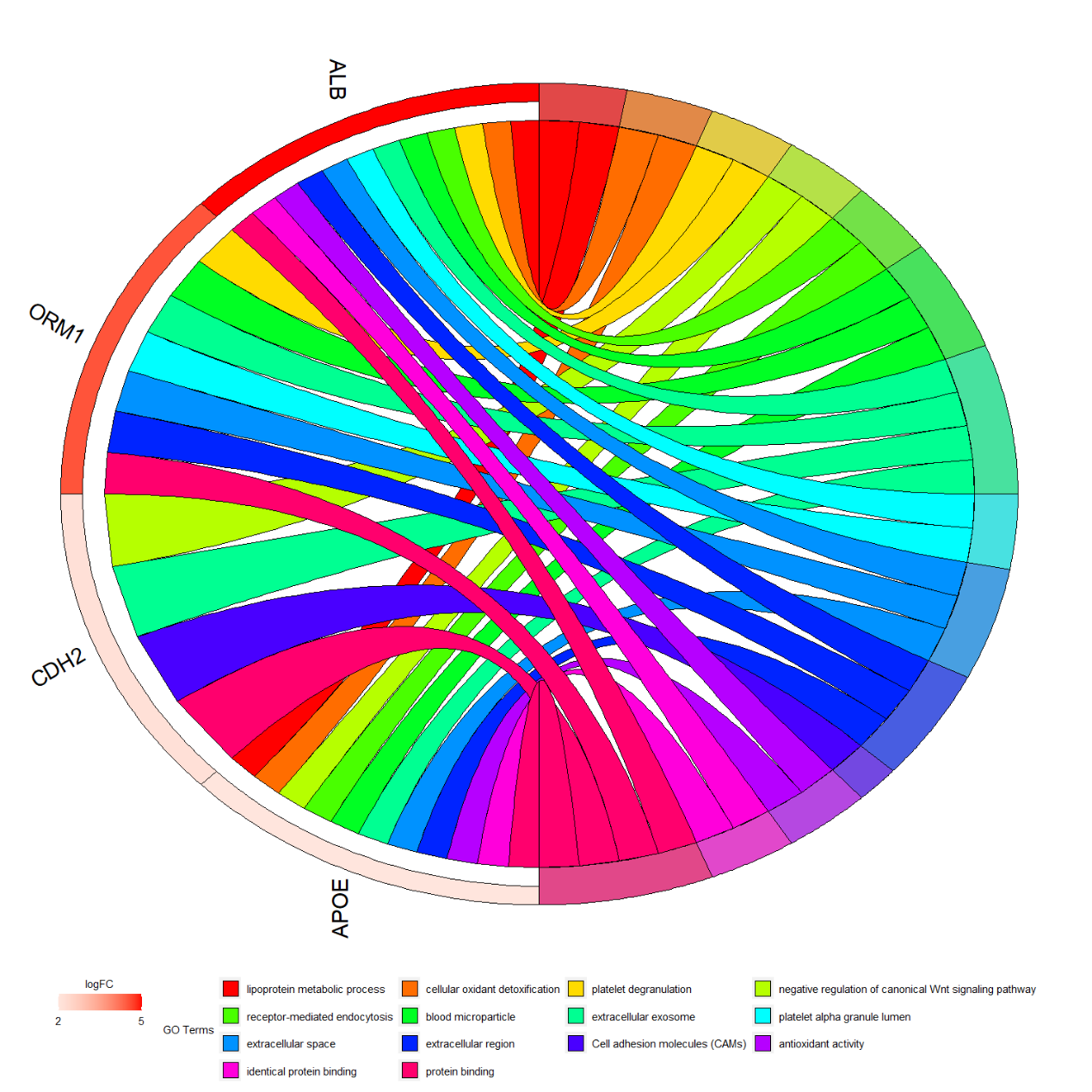


**Figure S5.** Hub genes enrichment Chord plot. Color of left semicircle indicates logFC of genes. Different colors in right semicircle represent different GO or KEGG terms.


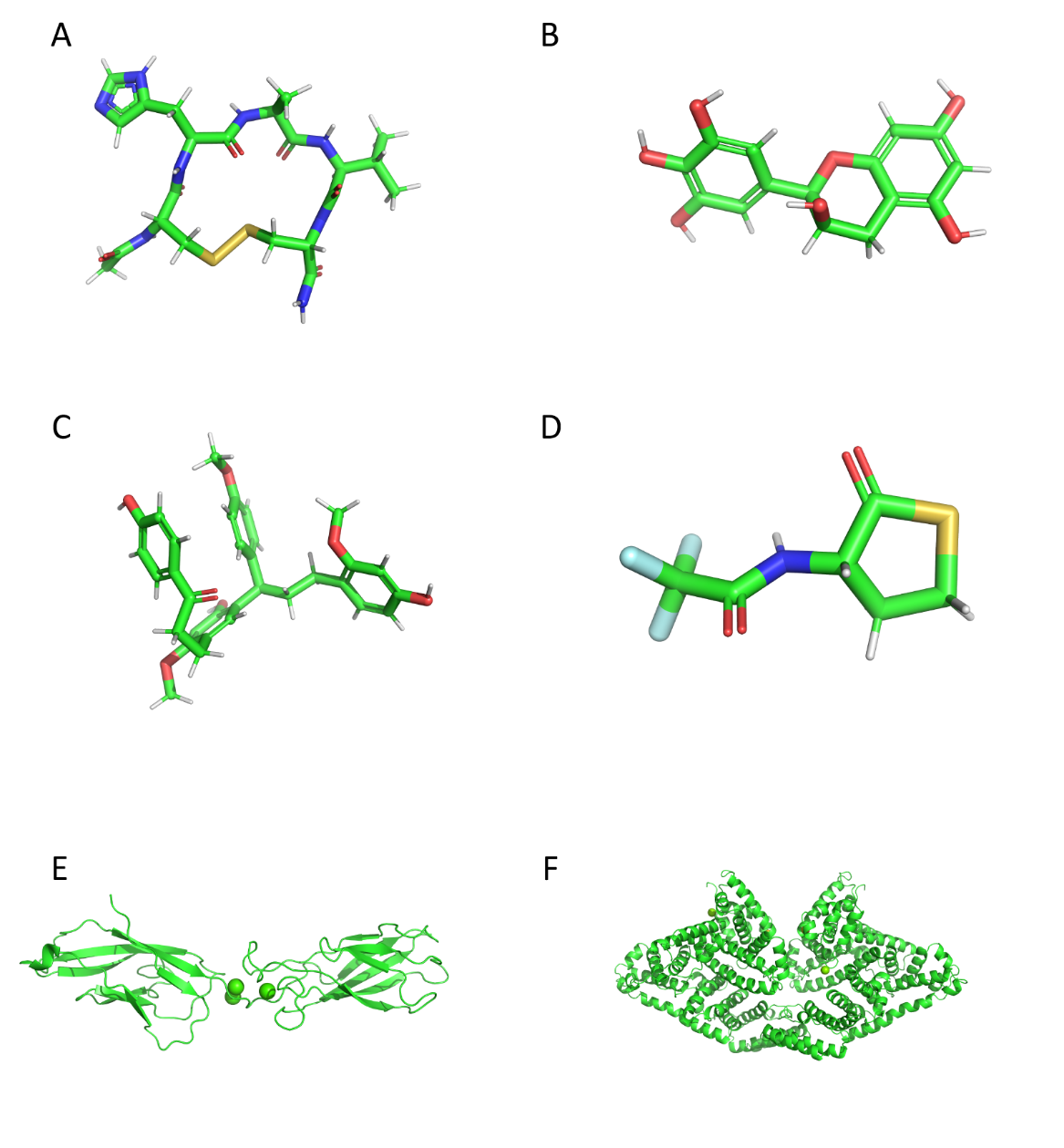


**Figure S6.** The structure of drugs and proteins. (A) ADH-1, (B) CHEMBL1945287, (C) Cochinchinenin C, (D) Epigallocatechin, (E) CDH2 protein structure, (F) ALB protein structure.


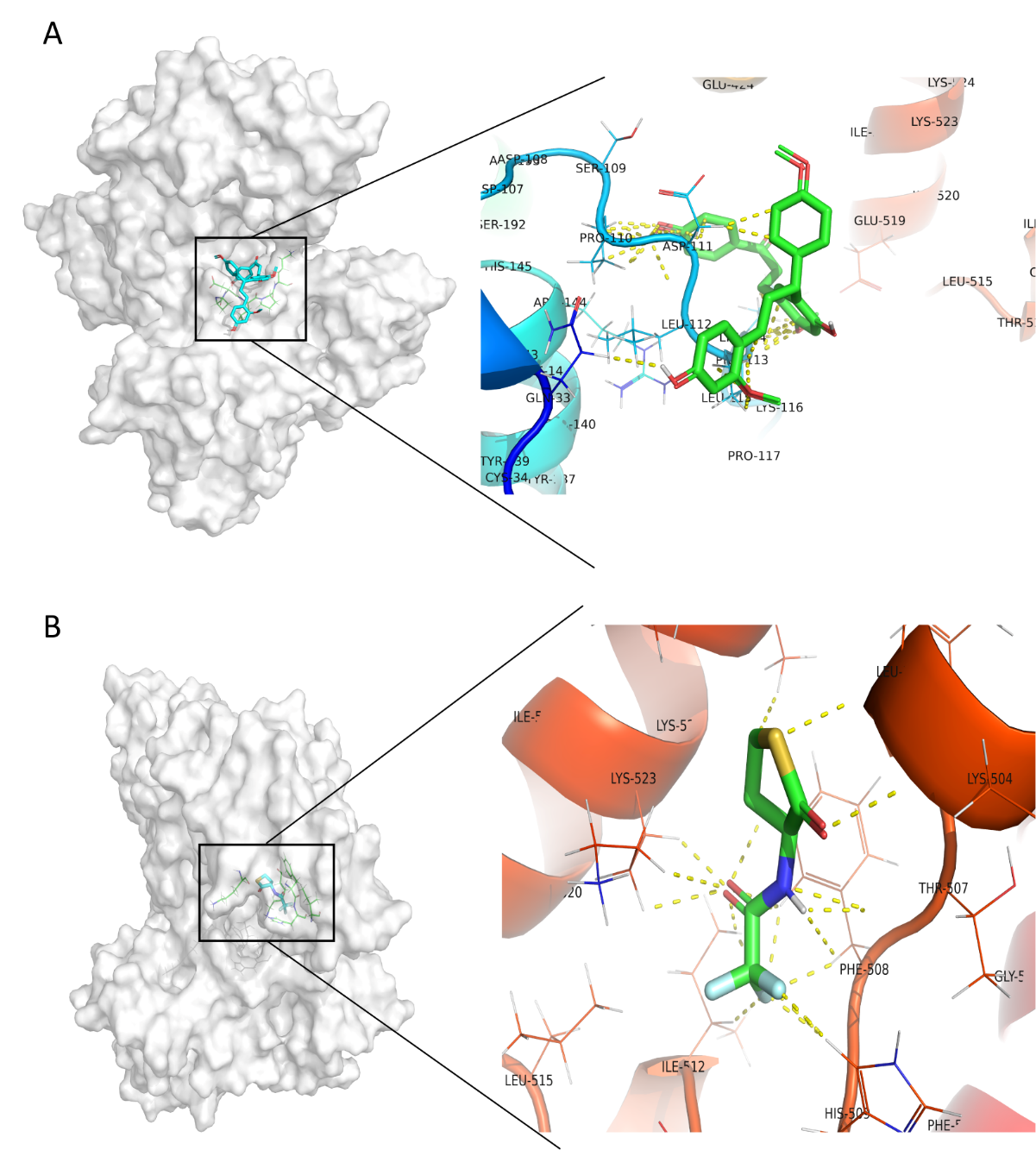


**Figure S7.** Protein-drug interactions between (A) ALB protein and Cochinchinenin C, (B) ALB protein and Epigallocatechin.
